# Supplementary material for: Effectiveness of Workplace Nutrition Programs on Anemia Status among Female Readymade Garment Workers in Bangladesh: A Program Evaluation
Source: Nutrients. 2019 Jun 3;11(6):1259. doi: 10.3390/nu11061259 (PMC6627420; doi:10.3390/nu11061259)
Supplement: Supplementary file 1 [file nutrients-11-01259-s001.pdf]

Supplementary file 1

Table S1. Composition of regular and fortified rice lunch meals served at readymade garment factories

	Servings per week × serving size (grams)		Average grams per day	
	**Regular lunch***	**Fortified lunch**	**Regular lunch**	**Fortified lunch**
**Rice***	375 g × 6 days	375 g × 6 days	375 g	375 g
**Lentil* †**	150 g × 6 days	150 g × 6 days	150 g	150 g
**Meat/poultry/fish**	48 g meat/poultry or 75 g fish × 3 days	90 g × 3 days minimum	29 g	45 g
**Egg**	50 g × 1.5	90 g × 1 day	13 g	15 g
**Total animal source foods**			42 g	60 g
**Green leafy vegetable**	-	90g × 6 days	0 g	90 g
**Other vegetables**	82 g (served with meat dish) or 135 g (no meat served) × 3 days	75 g (served with meat dish) × 2 days and 150 g (no meat served) × 3 days	54 g	50 g
**Total vegetables**			54 g	140 g
**Vegetable oil**	10 g × 6 days	10 g × 6 days	10 g	10 g
**Iron/folate supplement**	-	1	n/a	n/a

* Rice and lentils were provided in unlimited quantities to workers

† The thickness of the lentil was increased by doubling the content of lentils per volume of cooked lentil

Table S2. Comparison of the regular lunch menu and the fortified lunch menu according to the Dietary Guidelines for Bangladesh 2013 [1]*

	**DGB 2013 Recommendation [1]**		**Regular lunch menu**		**Fortified lunch menu**	
	**Per day**	**Applied to lunch meal****	**Amount provided**	**Meets recommendation**	**Amount provided**	**Meets recommendation**
**Energy, kcal**	2200	880	761	†	881	†
**Fat, % energy**	15-30%	15-30%	19%	Yes	20%	Yes
**Protein, % energy**	~10%	~10%	12%	Yes	15%	Yes
**Animal source foods, grams**	260	104	42	No	60	No
**Fruits, grams**	100	40	0	No	0	No
**Leafy vegetables, grams**	≥ 100	≥ 40	0	No	90	Yes
**Other vegetables, grams**	≥ 200	≥ 80	54	No	50	No
**Fruits & vegetables, grams**	≥ 400	≥ 160	54	No	140	No
**Sugar, grams**	≤ 20	≤ 8	0	Yes	0	Yes

*1. BIRDEM. Dietary Guidelines for Bangladesh; Dhaka, Bangladesh, 2013.

** Based on the assumption that the lunch meal would provide 40% of the total day's intake

†As rice and lentil were consumed without limit, the adequacy of energy provided by the menu could not be assessed. Kcal shown for regular and fortified menus used the estimated serving size of 375 grams cooked rice and 150 grams cooked lentil as shown in Table S1

Table S3. Estimated energy and nutrient content of regular and fortified lunch menu served in garment factories

Mean nutrient intake per day	Regular lunch	Fortified lunch + weekly iron/folate supplement *	Factory without lunch + twice weekly iron/folate supplement	Regular lunch	Fortified lunch + weekly iron/folate supplement *	Factory without lunch + twice weekly iron/folate supplement
	Average intake per lunch meal / per day			% Recommendation‡		
**Energy, kcal**	761	881	n/a	36	42	n/a
**Protein, g**	22	33	n/a	12 **	15 **	n/a
**Fat, g**	16	19	n/a	19 **	20 **	n/a
**Iron, mg**	2.7	22.6	17	5 / 9 †	38 / 77 †	29 / 58 †
**Zinc, mg**	3.1	8.2	n/a	32	84	n/a
**Folate, μg**	93	584	114	23	146	29
**Vitamin B12, μg**	0.68	2.13	n/a	28	89	n/a
**Vitamin A, μg RAE**	51	636	n/a	10	127	n/a

*Assumes Fortified lunch meal as summarized in Table S1, and substituting regular rice with multi-nutrient fortified rice and vitamin A-fortified vegetable oil, plus the daily equivalent of a once weekly iron/folate supplement (60 mg iron and 400 μg folate per weekly dose). The additional amount of iron/folate derived from the twice-weekly supplement without lunch is also shown.

**Expressed as recommended protein or fat content as % energy content

‡ Recommendations are the WHO/FAO Recommended Nutrient Intakes for micronutrients and recommendations for energy[2] and percent energy from protein and fat of the Dietary Guidelines for Bangladesh 2013[1]

† % RNI for iron shown for low / moderate bioavailability diets

Reference:

1. BIRDEM. Dietary Guidelines for Bangladesh; Dhaka, Bangladesh, 2013.
2. Food and Agriculture Organization of the United Nations; World Health Organization; United Nations University. Human energy requirements: Report of a Joint FAO/WHO/UNU Expert Consultation, Rome, Italy, 17-24 October 2001; FAO: Rome, 2004.

Table S4. Sensitivity analysis for replacement respondents at lunch meal intervention factory

Indicators	Before replacement	After replacement	Overall	p-value
Respondents age, mean ± SD	27.1 ± 4.9	28.4 ± 5.3	27.8 ± 5.1	0.69
Wealth index, n(%)				
Lowest	90 (17)	93 (18.2)	183 (17.6)	
Second	99 (18.7)	91 (17.8)	190 (18.3)	
Middle	97 (18.3)	105 (20.6)	202 (19.4)	0.815
Fourth	110 (20.8)	103 (20.2)	213 (20.5)	
Highest	134 (25.3)	118 (23.1)	252 (24.2)	
Marital status, n (%)				
Married	426 (80.4)	422 (82.8)	848 (81.5)	
Unmarried	65 (12.3)	51 (10)	116 (11.2)	0.502
Divorced/ separated/widow	39 (7.4)	37 (7.3)	76 (7.3)	
Education, n (%)				
No	72 (13.6)	70 (13.7)	142 (13.7)	0.947
Yes	458 (86.4)	440 (86.3)	898 (86.4)	
Place of residence, n (%)				
Rural	97 (18.3)	97 (19)	194 (18.7)	0.766
Urban	433 (81.7)	413 (81)	846 (81.4)	
Religion, n(%)				
Islam	495 (93.4)	482 (94.5)	977 (93.9)	
Hindu	33 (6.2)	27 (5.3)	60 (5.8)	0.697
Christian	2 (0.4)	1 (0.2)	3 (0.3)	
Household ownership, n (%)				
Own house	54 (10.2)	56 (11)	110 (10.6)	0.678
Rented house/ shared	476 (89.8)	454 (89)	930 (89.4)	
Working overtime, n (%)				
No	19 (3.6)	26 (5.1)	45 (4.3)	0.231
Yes	511 (96.4)	484 (94.9)	995 (95.7)	

Continuous variables are presented by mean ± SD and categorical variables are presented as the percentage of participants (%).

Table S5. DID analysis reporting both replacement and non-replacement participant results: Effect of intervention on anemia reduction (% of women with any anemia)

	Baseline			Endline			Unadjusted DID (E-B)	Adjusted DID[1]
Indicator	I	C	B=Diff (I-C)	I	C	E=Diff (I-C)		
With replacement participants								
Lunch meal (A vs B)	60.7	33.2	27.5	36.9	41.2	-4.3	-31.8***	-32.4***
Non meal (C vs D)	47.9	36.3	11.6	41.5	41.8	-0.3	-11.9**	-11.6**
Nonreplacement participants								
Lunch meal (A vs B)	63.8	33.3	30.5	35.1	41.1	-6.0	-36.5***	-37.0***
Non meal (C vs D)	48.3	35.9	12.4	39.4	39.8	-0.4	-12.8*	-12.0**

Abbreviation: I: intervention; C: control. Indicator variable anemia changes are presented as the percentage points (%). Model 1: adjusted for marital status, asset index, household ownership, overtime work hours per month, baseline anemia difference and intracluster correlation (ICC); *** p <0.0001; **p <0.01.

Table S6. Changes of characteristics over the baseline to endline survey

Variables	Lunch meal						Non-meal					
	Baseline	Endline	p	Baseline	Endline	p	Baseline	Endline	p	Baseline	Endline	p
	I (n=326)	I (n=306)		C (n=328)	C (n=328)		I (n=328)	I (n=328)		C (n=328)	C (n=328)	
Asset quintile, n (%)												
Poorest	61 (18.7)	65 (21.2)		93 (28.4)	105 (32)		54 (16.5)	47 (14.3)		50 (15.2)	60 (18.3)	
Poorer	58 (17.8)	60 (19.6)		86 (26.2)	61 (18.6)		54 (16.5)	48 (14.6)		70 (21.3)	75 (22.9)	
Middle	63 (19.3)	64 (20.9)	NS	61 (18.6)	63 (19.2)	0.03	77 (23.5)	60 (18.3)	NS	59 (18)	66 (20.1)	NS
Richer	59 (18.1)	59 (19.3)		58 (17.7)	50 (15.2)		62 (18.9)	80 (24.4)		69 (21)	59 (18)	
Richest	85 (26.1)	58 (19)		30 (9.1)	49 (14.9)		81 (24.7)	93 (28.4)		80 (24.4)	68 (20.7)	
Overtime hours in a month, Mean (SD)	33.3 (10.5)	49.6 (17)	<0.001	41.1 (9.3)	45.5 (8.5)	<0.001	44.6 (19.5)	38.6 (20.6)	<0.001	47.4 (12)	44.1 (12)	<0.001
Total Income USD, Median (IQR)	100 (88, 108)	118 (101, 131)	<0.001	108 (100, 113)	113 (105, 119)	<0.001	104 (92, 118)	102 (92, 113)	NS	109 (98, 116)	113 (104, 124)	<0.001
Total Household Expenditure, Median(IQR)	94 (85, 106)	113 (100, 128)	<0.001	100 (88, 113)	108 (100, 115)	<0.001	101 (88, 114)	100 (89, 113)	NS	106 (93, 113)	113 (100, 119)	NS

Abbreviation: I: intervention; C: control; IQR: interquartile range. Continuous variables are presented by mean ± SD and categorical variables are presented as the percentage of participants (%).
